# Homicidal ideation and psychiatric comorbidities in the inpatient adolescents aged 12–17

**DOI:** 10.3389/fpsyt.2022.933524

**Published:** 2022-11-16

**Authors:** Ching-Fang Sun, Zeeshan Mansuri, Chintan Trivedi, Ramu Vadukapuram, Abhishek Reddy

**Affiliations:** ^1^Department of Psychiatry, Virginia Tech Carilion School of Medicine, Roanoke, VA, United States; ^2^Department of Psychiatry, Boston Children’s Hospital, Harvard Medical School, Boston, MA, United States; ^3^Department of Psychiatry, Texas Tech University Health Science Center at Permian Basin, Odessa, TX, United States; ^4^Department of Psychiatry, The University of Texas Rio Grande Valley, Harlingen, TX, United States

**Keywords:** homicide, psychiatric comorbidity, adolescents, depression, bipolar disorder, anxiety

## Abstract

**Objectives:**

Adolescents with a homicidal tendency is a growing concern in the United States. Studies in the past have showcased the relationship between homicidal ideation (HI) and psychiatric illnesses, but very limited information is available on the adolescent and inpatient population. We aim to evaluate the prevalence of demographic characteristics and psychiatric disorders in adolescents with and without HI.

**Materials and methods:**

Adolescent (age 12–17) population admitted to the hospital with the diagnosis of homicidal ideation was identified from the 2016–2018 National Inpatient Sample Dataset (NISD). Patients without HI were defined as the control group. The prevalence of psychiatric comorbidities between the groups was compared by applying the Rao-Scott adjusted chi-square test. We used multivariable logistic regression to generate odds ratio (OR) of homicidal ideation as an outcome; we adjusted age, sex, race, socioeconomic status, substance use disorders, alcohol use disorders, and psychiatric comorbidities.

**Results:**

A total of 18,935 patients (mean age: 14.5) with HI diagnosis were identified in this study. Majority of the patients were male subjects in the HI group compared to the control group (58.7 vs. 41.2%, *p* < 0.001). Racially, HI was more prevalent in white race (56.0 vs. 52.6%, *p* < 0.001) and black race (22.3 vs. 17.8%, *p* < 0.001), compared to Hispanic race (14.9 vs. 21.3%, *p* < 0.001). Major depression (Odds ratio [OR]: 2.66, *p* < 0.001), bipolar disorder (OR: 3.52, *p* < 0.001), anxiety disorder (OR: 1.85, *p* < 0.001), ADHD, and other conduct disorders (OR: 4.01, *p* < 0.001), schizophrenia (OR: 4.35, *p* < 0.001) are strong predictors of HI. Suicidality was prevalent in 66.9% of patients with HI.

**Conclusion:**

We found a higher prevalence of psychiatric illnesses such as depression, anxiety, and bipolar disorder in adolescents with homicidal ideation in the inpatient setting. White and black races were more prevalent in patients with homicidal ideation. Further large-scale longitudinal research studies are warranted to establish the correlation between psychiatric disorders and homicidal ideation among adolescents.

## Introduction

Though the adolescent legal offense case rates in the US are generally trending down, the number of juvenile criminal homicides has conversely increased by 49% in the recent 5 years, while most offenders are older than 16 (6%), male subjects (89%), and non-Caucasian (72%) (1). The number of murder offenders peaks at the age group of 20–29, but the age group of 17–19 has the highest number (2). Homicidal events are a growing concern in the US. Homicidal events, especially those with firearms, illicit the publics’ fear about personal safety; decrease satisfaction with law enforcement and the trust of others (3). Also, violence is a form of financial burden to society. A single homicide could cost nearly $4 million (4).

Homicidal ideation in the adolescent population deserves public attention. Adolescents endorse the tendency toward rewards approach behavior with increased emotion reactivity and limited impulse control due to immature neurodevelopmental status (5–7). However, they might have to undergo the same legal process as adults. In most states in the US, the maximum age of juvenile court jurisdiction is age 17, but murder and serious violent felony cases will be excluded from juvenile court (8). Furthermore, violent and chronic young offenders are very likely to become adult delinquents (9, 10). Of note, homicidal ideation is not only related to homicidal events, but various kinds of criminal activities with higher severity (10). As a result, early identification and intervention is a reasonable, cost-effective strategy to protect both the youth and society.

Previous research has shown the correlation between HI and psychiatric illnesses in adolescents, with the evidence that students with mental health issues were arrested at a rate of 2.9 times more than their healthy counterparts (11, 12). Juvenile offenders with mental illness also have a greater risk of re-offending (13). Childhood maltreatment, conduct disorder, attention-deficit hyperactivity disorder, mood disorder, tic disorder, and abnormal neuropsychiatric findings such as learning disabilities have been proven to increase the odds of comorbid HI (12, 14–16). HI is also well-known to harbor depression and suicidal ideation (SI) (17).

Despite evidence demonstrating substantial mental health conditions predisposing to HI in adolescents, minimal information is available on the adolescent inpatient population, the most vulnerable group needs a higher level of care. Our study aims to evaluate the difference in demographic characteristics and comorbid psychiatric disorders in adolescents with and without HI in a nationwide prospect.

## Material and methods

### Methods

We reviewed patient records from the Nationwide Inpatient Sample (NIS) dataset during 2016–2018. The NIS database is an administrative dataset from the Healthcare Cost and Utilization Project (HCUP) and Agency for Healthcare Research and Quality (AHRQ) (17). NIS is released annually and contains information on over 150 patient and hospital-level data elements, such as diagnoses, procedures, patient demographics (e.g., sex, age, race, median income), and discharge disposition, primary payer, length of stay, and hospital characteristics (bed size, location, and teaching status). The NIS provides weights (the trend weight prior to the year 2011 and the discharge weight after the year 2011) that allow nationally representative estimates. As NIS excludes data elements that could directly or indirectly identify individuals, this study was exempt from review by the institutional review board. Also, each record contains primary (indication for hospital admission) and secondary diagnosis information based on the International Classification of Diseases, Clinical Modification/Procedure Coding System (ICD-10-CM). Diagnoses were aggregated according to the ICD-10 code provided in Clinical Classification Software groupings of psychiatric and substance-related disorders.

### Data collection

The study group was defined as adolescent aged 12–17 with homicidal ideation (ICD-10 code: R45.850) by applying the ICD-10 code provided in the NIS dataset. Adolescent aged 12–17 without homicidal ideation (ICD-10 code: R45.850) were defined as the control group. We collected baseline demographic data for both the study and control group. Further, we collected data on psychiatric and substance use disorder comorbidities based on the clinical classification software grouping provided on the HCUP website.

### Statistical analysis

We calculated mean and standard error to present continuous data and percent for categorical data. All tests were two-sided. A *p*-value of less than 0.05 was considered statistically significant. The prevalence of psychiatric comorbidities between the groups was compared by applying Rao-Scott adjusted chi-square test. Multivariable logistic regression was performed with homicidal ideation as an outcome; we adjusted age, sex, race, socioeconomic status, substance use disorders, alcohol use disorders, and psychiatric comorbidities. All statistical analyses were performed by using the SPSS version 26.0 software for Windows (IBM Software, Inc., Armonk, NY, USA).

## Results

**FIGURE 1:**
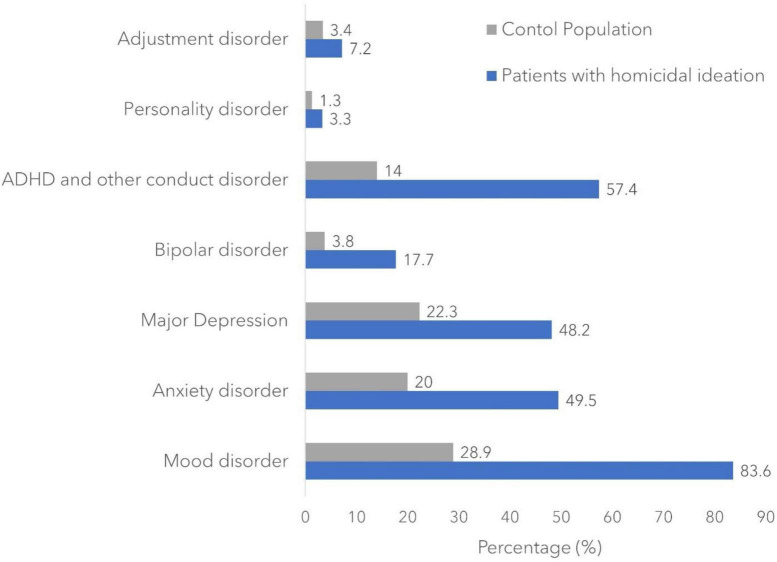
Incidence rate of psychiatric comorbidities among the study population.

The baseline characteristics of the study group and control group are shown in Table 1. A total of 18,935 patients (mean age: 14.5 years) with HI diagnosis were identified with a male predominant pattern (58.7 vs. 41.2%, *p* < 0.001). Racially, HI was more prevalent in white race (56.0 vs. 52.6%, *p* < 0.001) and black race (22.3 vs. 17.8%, *p* < 0.001) compared to Hispanic race (14.9 vs. 21.3%, *p* < 0.001). Patients with HI were more likely to be covered by Medicare or Medicaid insurance (59.4 vs. 50.6%, *p* < 0.001) with a lower household income (34.4 vs. 30.7% in the lowest 25% of household income, *p* < 0.001). No significant difference was found in the rural or urban area, whereas HI was more prevalent in hospitals in the Midwest and southern region of the US (33.8 vs. 23.9%; 43.4 vs. 39.9%). All the psychiatric comorbidities included in our study were more prevalent in patients with HI (Figure 1) with a *p*-value less than 0.001. Suicidality including suicidal ideation and suicide attempt was prevalent in 66.9% of patients with HI.

**TABLE 1:** Demographic characteristics of the patients (age 12–17) with and without homicidal ideation.

	Patients with HI *N* = 18,935	Patients without HI *N* = 1,645,629	*P-value*
Age, mean, SE	14.49 (0.04)	14.86 (0.01)	<0.001
Sex			<0.001
Male	58.7	41.2	
Female	41.3	58.8	
Insurance			<0.001
Medicare/Medicaid	59.4	50.6	
Private insurance	35.6	43.0	
Self-pay/No Charge/Other	6.5	5.1	
Location/teaching status of hospital			0.57
Rural	4.1	4.5	
Urban	95.9	95.5	
Region of hospital			<0.001
Northeast	12.9	16.2	
Midwest	33.8	23.9	
South	43.4	39.9	
West	10.0	20.0	
Race			<0.001
White	56.0	52.6	
Black	22.3	17.8	
Hispanic	14.9	21.3	
Asian or Pacific Islander	1.5	2.6	
Native American	1.1	0.9	
Other	4.3	4.9	
Median household income			<0.001
0–25th Median	34.4	30.7	
25th-50th Median	27.6	25.1	
51st-75th Median	22.3	23.5	
76th-100th Median	15.6	20.7	

The results of the multivariable analysis are shown in Table 2. In the multivariable analysis, major depression (OR: 2.66, *p* < 0.001), bipolar disorder (OR: 3.52, *p* < 0.001), anxiety disorder (OR: 1.85, *p* < 0.001), ADHD, other conduct disorders (OR: 4.01, *p* < 0.001), and schizophrenia (OR: 4.35, *p* < 0.001) were strong predictors of the outcome. No association was found between personality disorders and homicidal ideation (*p* = 0.27).

**TABLE 2:** Multivariable analysis for predictors of homicidal ideation.

	Odds ratio (95% confidence interval)	*P-value*
Major depression	2.66 (2.37-2.99)	<0.001
Bipolar disorder	3.52 (3.07-4.05)	<0.001
Personality disorder	1.15 (0.90-1.46)	0.27
Anxiety disorder	1.85 (1.66-2.06)	<0.001
ADHD and other conduct disorder	4.01 (3.67-4.38)	<0.001
Schizophrenia	4.35 (3.79–4.98)	<0.001

## Discussion

### Demographic features

The demographic feature of adolescents with HI in our study demonstrates a propensity which is similar to adolescents being arrested for murder and non-negligent manslaughter: male predominant (58.7 vs. 41.2%, *p* < 0.001), white (56.0 vs. 52.6%, *p* < 0.001), and black races (22.3 vs. 17.8%, *p* < 0.001) more than other races. According to the Office of Juvenile Justice and Delinquency Prevention data, 89% of minor offenders charged with murder and non-negligent manslaughter are male subjects; 47% of them are white; and 50% are black (18).

Homicidal behavior is well-known to be more prevalent in male subjects. We also want to highlight that female subjects in our study were more likely to present with HI compared to the real-world criminal statistic results. Previous studies explained our findings with evidence that female homicide offenders are more likely to have a diagnosis of mental illness (19). With a higher prevalence of mental illness, they are more likely to be hospitalized and thus included in our study.

Higher prevalence of HI in white adolescents may result from racial/ethnic disparity in health services. White adolescents are more likely to be recognized with HI because healthcare is more approachable. The racial demographic data in adolescents with HI are also comparable to adolescent suicidality. More white adolescents endorsed SI, while more black adolescents attempted suicide (20). Black adolescents had a higher rate of suicide attempts compared to their white counterparts. The ideation-to-action model of suicidal behavior could possibly explain this phenomenon (21). We assume that the same model could be applied to homicidal behavior. Not every aggression consequently develops into homicidal ideation. Repetitive exposure to trauma and violence cause habituation of aggression which later could escalate into homicidal ideation. Black adolescents are more likely to experience inequitable sociopolitical contexts (e.g., police hostility) and microsystem (e.g., unsafe neighborhoods). Due to the increased psychosocial burden, the incubation period for aggression to escalate might be shorter and thus less likely to be detected. Hispanic adolescents with HI might be underdiagnosed due to similar reasons (22). Previous studies showed that black people are more likely to be both the offender and victim of homicide/assault (23). The necessity of self-defense and the violent drive from the fear of being killed should also be taken into consideration.

Although we could not obtain the exact family social economic status of our patient population, our data showed that patients with HI were more likely to be covered by public insurance (Medicare or Medicaid) with a lower household income. The insurance type and income could at least provide a glimpse of the undesirable financial environment in certain patient populations. Low social economic status indicates stressful life situations, higher rate of behavior issues, and psychopathology (24, 25). Adolescents from a household struggling financially are also more likely to be a victim of bully (26). Adverse childhood events including school bullying are significantly related to the development of HI (27–29).

### Depression, bipolar, anxiety, and homicidal ideation

Neuroimaging shows that depressive symptoms and impulsivity in children and adolescents share common features, including reduced cortical thickness in the ventromedial prefrontal cortex and medial orbitofrontal cortex. Moreover, impulsivity is specifically associated with reduced cortical thickness in the lateral prefrontal region and frontal pole (30). Children and adolescents with bipolar disorder and aggressive behavior also have cortical thinning in prefrontal and parietal cortices (31). Additionally, depression, bipolar, anxiety, and aggression shared many common physiological characteristics including dysregulated hypothalamic–pituitary–adrenal (HPA) axis, abnormal serotonin system, and chronic inflammation (32–34).

Anxiety increases indirect aggression like anger and hostility, also physical aggression in the context of high impulsivity in adolescents (35, 36). As a part of early programming for humans to survive an aversive situation, anxiety is associated with a fight and flight response and thereby coexists with aggression as an adaptive reaction toward threats in nature. Anxiety and aggression have multiple overlapping brain pathways involving the HPA axis, arginine vasopressin, gamma-aminobutyric acid, testosterone, and serotonin (37). The fact that admitting anxiolytics decreases aggression also supports the anxiety-aggression correlation (38). These evidence explained the higher prevalence of anxiety in adolescents with HI.

### Attention-deficit/hyperactivity disorder, conduct disorder, personality disorders, and homicidal ideation

Attention-deficit/hyperactivity disorder and conduct disorder were associated with homicidal ideation, so as personality disorders (39). Not every ADHD child endorsed the tendency of violence. However, ADHD was known to be related to impulsive and risky decision making (40). Conduct disorder is a strong predictor of antisocial personality disorder later in life, while both are related to increased risk of HI and being an offender of homicidal events (10, 41).

Previous studies showed increased HI in patients with personality disorders which include antisocial personality disorder, borderline personality disorder, paranoid personality disorder, obsessive-compulsive personality disorder, and avoidant personality disorder (41). Among all the personality disorders, the literature review showed a significant correlation between antisocial personality disorder and HI (41–43). We did not find personality disorders as a strong predictor of HI in the adolescent population, which might be a result of underdiagnosis. Considering the similarity of certain personality features and normal adolescent behavior, mental health professionals tend not to diagnose personality in adolescents (44). Another reason for clinicians to avoid diagnosing adolescents with personality disorders is to avoid stigmatizing patients with a long-lasting, treatment-resistant condition (44, 45).

### Suicidal ideation and homicidal ideation

We found that HI was commonly present with SI, which was also seen in previous studies. The co-occurrence of HI and SI may be explained by the pathophysiology shared by depression, bipolar, and anxiety. It could also be illustrated from a psychoanalytic standpoint. According to Karl Menninger, there are three wishes binding together to push a person to suicide: the wish to kill, the wish to be killed, and the wish to die (46). Thus, we can postulate that there is a relationship between SI and HI.

The projective-introjective cycling of aggressive impulse is another explanation of HI accompanied by a suicidal wish (47). Children’s and adolescents’ concept of self is not completely formed. Thus, they are more likely to experience blurring boundaries of self and others, especially with those raised by abusive parents (47). While experiencing chronic stress and emotional suffering, they would have difficulty attributing the psychological content. The direction of aggression thereby vacillates between self and others. In other words, they become alternatively suicidal and homicidal (48).

A recent study on juvenile delinquents showed that suicidal ideation was correlated with certain features in psychopathy: carefree non-planfulness, blame externalization, and rebellious non-conformity (49). Thereby, sharing similar behavioral and lifestyle features of psychopathy might explain the co-occurrence of HI and SI.

### Limitations

A limitation of our study lies in the fact that we only include the inpatient population. Besides depression and anxiety, homicidal ideation is also prevalent in adolescents with an autism spectrum disorder (50). While most patients with autism spectrum disorders are more likely to be managed outpatient, they might be excluded from our study. Adolescents with mild personality traits who are predisposed to HI are even less likely to be assessed in an inpatient medical setting.

Some other limitations are derived from the nature of our database. The ICD coding system was established mainly for billing purposes. Thus, the ICD codes may not truly reflect patients’ overall situation. It is also not possible for us to see the severity of HI and its context. Additionally, the NISD did not provide vital information that would significantly affect adolescents’ mental health, such as family history, attachment style, early-childhood experience, history of emotional/physical/sexual abuse, or neglect. Last but not least, our result only demonstrated the propensity of correlation between HI and diagnoses but not the causality.

## Conclusion

Our study provides updated demographic data of adolescents with HI in an inpatient setting: male subjects, the white race was more prevalent in the homicidal group. We also found a higher prevalence of psychiatric illnesses such as depression, anxiety, and bipolar disorder in adolescents with HI. Our result is also consistent with previous findings that HI is highly comorbid with SI. Further large-scale longitudinal research studies are warranted to establish the correlation between psychiatric disorders and homicidal ideation among adolescents.

## Data availability statement

Publicly available datasets were analyzed in this study. This data can be found here: https://www.hcup-us.ahrq.gov/.

## Ethics statement

Ethical review and approval were not required for the study on human participants in accordance with the local legislation and institutional requirements. Written informed consent for participation was not required for this study in accordance with national legislation and institutional requirements.

## Author contributions

AR, ZM, and CT contributed to the idea, writing, editing, and reviewing of the manuscript. C-FS and RV contributed to the writing, editing, and reviewing of the manuscript. All authors contributed to the article and approved the submitted version.
